# Growth rate regulated genes and their wide involvement in the *Lactococcus lactis *stress responses

**DOI:** 10.1186/1471-2164-9-343

**Published:** 2008-07-21

**Authors:** Clémentine Dressaire, Emma Redon, Helene Milhem, Philippe Besse, Pascal Loubière, Muriel Cocaign-Bousquet

**Affiliations:** 1 Université de Toulouse; INSA, UPS, INP, LISBP, 135 Avenue de Rangueil, F-31077 Toulouse, France; 2 UMR5504, UMR792 Ingénierie des Systèmes Biologiques et des Procédés, CNRS, INRA, 31400 Toulouse, France; 3 Laboratoire de Biologie et Biotechnologies marines, IBFA, UMR100 IFREMER, Physiologie et Ecophysiologie des Mollusques Marins, Université de Caen Basse-Normandie, Esplanade de la Paix, F14032 Caen Cedex, France; 4 Université de Toulouse ; INSA ; F-31077 Toulouse, France; 5 CNRS ; IMT (Institut de Mathématiques de Toulouse) ; F-31077 Toulouse, France

## Abstract

**Background:**

The development of transcriptomic tools has allowed exhaustive description of stress responses. These responses always superimpose a general response associated to growth rate decrease and a specific one corresponding to the stress. The exclusive growth rate response can be achieved through chemostat cultivation, enabling all parameters to remain constant except the growth rate.

**Results:**

We analysed metabolic and transcriptomic responses of *Lactococcus lactis *in continuous cultures at different growth rates ranging from 0.09 to 0.47 h^-1^. Growth rate was conditioned by isoleucine supply. Although carbon metabolism was constant and homolactic, a widespread transcriptomic response involving 30% of the genome was observed. The expression of genes encoding physiological functions associated with biogenesis increased with growth rate (transcription, translation, fatty acid and phospholipids metabolism). Many phages, prophages and transposon related genes were down regulated as growth rate increased. The growth rate response was compared to carbon and amino-acid starvation transcriptomic responses, revealing constant and significant involvement of growth rate regulations in these two stressful conditions (overlap 27%).

Two regulators potentially involved in the growth rate regulations, *llrE *and *yabB*, have been identified. Moreover it was established that genes positively regulated by growth rate are preferentially located in the vicinity of replication origin while those negatively regulated are mainly encountered at the opposite, thus indicating the relationship between genes expression and their location on chromosome. Although stringent response mechanism is considered as the one governing growth deceleration in bacteria, the rigorous comparison of the two transcriptomic responses clearly indicated the mechanisms are distinct.

**Conclusion:**

This work of integrative biology was performed at the global level using transcriptomic analysis obtained in various growth conditions. It raised the importance of growth rate regulations in bacteria but also participated to the elucidation of the involved mechanism. Though the mechanism controlling growth rate is not yet fully understood in *L. lactis*, one expected regulatory mechanism has been ruled out, two potential regulators have been pointed out and the involvement of gene location on the chromosome has also been found to be involved in the expression regulation of these growth related genes.

## Background

Natural free living bacteria, as well as industrially exploited strains, have to face many challenging situations such as chemical (pH, salinity) and physical (temperature) changes of the environment or nutrient deprivation. Pooled under the generic term of stress, these environmental challenges share a common feature: they all affect the cellular growth rate. Many studies, mostly using transcriptomic approach, have exhaustively described the responses to various stresses in different bacteria [1-4]. However these studies performed in batch cultures do not allow the growth rate response to be specifically described because influences of the specific stress and the growth rate are superimposed. The exclusive cellular response associated to growth rate changes can be achieved through chemostat cultivation. This fermentation mode enables the growth rate to be changed by the dilution rate monitoring without modifying environmental or nutritional parameters [5]. Transcriptomic analysis in chemostat have been performed mainly in *Saccharomyces cerevisiae *[6-10] and the specific response associated to growth rate modifications has been characterized. The authors demonstrated that a large part of the genome expression, ranging between 27 and 50%, was affected by the growth rate [7-9]. Growth rate influence is also major in bacteria and Egli and co-workers [11] demonstrated in chemostat culture that the sensitivity of *Escherichia coli *to stress was dependent on the specific growth rate. However a complete analysis combining chemostat cultivation and transcriptomic approach to clearly study the growth rate influence at the global level has never been performed in bacteria.

Currently, the existence of a general mechanism activated by the bacteria to cope with any stressful condition slowly develops and a rising hypothesis considers this biological process to be the stringent response [12,13]. Chang and colleagues even go ahead by clearly proposing stringent response to be triggered by the cells when growth rate decreases independently of this decrease origin [14]. Stringent response is a well known mechanism involved in bacteria mediated by ppGpp (see [13] for review), however its strict comparison to growth rate response has never been reported, the unique growth rate study being available for *S. cerevisiae *[7-9], a micro-organism lacking the stringent mechanism. Thus, the assumption that the regulation involved in the growth rate control may be identical to the stringent response is emerging though no clear evidence is available in the literature.

In order to characterize growth rate effect in the model of lactic acid bacteria,*Lactococcus lactis*, we have performed chemostat cultures at various growth rates and analysed the cellular response to this growth rate changes by transcriptomic approach. The transcriptomic response was then compared to the one obtained when the growth rate was modified by stress (carbon and amino acid starvations) in batch cultures. Lastly the norvaline response, a chemical inductor previously used in *Bacillus subtilis *[15] to induce the stringent response, was characterised and compared to the growth rate response.

## Methods

### Strain and growing conditions

The strain *Lactococcus lactis *IL1403, whose genome is available [16] was grown in anaerobic chemostat cultures (under nitrogen atmosphere) on a chemically defined medium. The medium composition (Table 1) is derived from the CDM medium [17,18] with a ten-fold reduction of Branched-Chain Amino Acid (BCAA) concentrations *i.e*., valine, leucine, isoleucine. Continuous cultures were performed in a 0.5 L fermentor (Verrerie Wagner, Toulouse, France) maintained at a constant temperature of 30°C and under nitrogen atmosphere. The pH was regulated at 6.6 by automatic addition of KOH (10 N). Four different growth rates have been studied, namely 0.09, 0.24, 0.35 and 0.47 h^-1^. For each steady-state studied, samples have been harvested in at least quadruplicate with a minimum delay of five doubling time between each sampling.

**Table 1:** Composition of the culture medium.

Product	Concentration (g/L)	Product	Concentration (g/L)
Glucose	20	Isoleucine	0.025
Sodium acetate	1	Leucine	0.05875
Ammonium citrate	0.6	Valine	0.04125
		
KH_2_PO_4_	3 (9)	Cysteine	0.17
		
K_2_HPO_4_	2,5 (7,5)	Phenylalanine	0.28

MgCl_2_, 6H_2_O	0.2	Tyrosine	0.29
FeSO_4_, 7H_2_O	0.011	Adenine	0.05
CaCl_2_, 2H_2_O	0.05	Guanine	0.05
ZnSO_4_, 7H_2_O	0.005	Uracil	0.05
COCl_2_, 6H_2_O	0.0025	Xanthin	0.05

Alanine	0.24	P-aminobenzoic acid	0.01
Arginine	0.12	Biotin	0.01
Asparagine	0.34	Cyano-cobalamine (B12)	0.001
Glutamine	0.51	Folic acid	0.01
Glycine	0.17	Inosine	0.025
Histidine	0.11	Nicotinic acid	0.001
Lysine	0.35	Orotic acid	0.005
Methionine	0.12	Ca panthotenate	0.001
Proline	0.68	Pyridoxamine	0.005
Serine	0.34	Pyridoxin (B6)	0.002
Threonine	0.23	Riboflavin (B2)	0.001
Tryptophan	0.05	Thiamine	0.001
		D, L-6,8-thioctic acid	0.0025
		Thymidine	0.025

The lines separating the different products indicate those that are dissolved together. Values into brackets in front of KH_2_PO_4 _and K_2_HPO_4 _indicate the required concentrations to obtain a buffered media for pre-cultures

To study isoleucine starvation response, *L. lactis *was grown in batch cultures in similar medium (Table 1). Cultures were grown under nitrogen atmosphere in a 2 L fermentor (Setric Génie Industriel, Toulouse, France) at 30°C with an agitation speed of 250 rpm. The pH was maintained at 6.6 by automatic addition of KOH 10 N. To study the stringent response, cells were grown similarly in batch cultures in a medium containing two-fold increase of BCAA compared to the medium described in Table 1 and norvaline was added at a final concentration of 10 g.l^-1 ^during the exponential growth phase.

### Analytical methods

Bacterial growth was estimated by spectrophotometric measurements at 580 nm (Hitachi U1100, 1 Unity of absorbance is equivalent to 0.3 g·L^-1^). Glucose, lactate, acetate and ethanol concentrations were measured by high pressure liquid chromatography (Hewlett Packard 1050; Waters 717 autosampler; Biorad microguard; H+ column Biorad HPX87H; Perkin Elmer LC90Bio UV detector; Hewlett Packard 1047A refractometer). Amino-acids concentrations in culture supernatants were measured via the AminoQuant HP1090 system (column: Hypersil-AA-ODS, 200*2.1 mm, 5 μm, Agilent).

### Transcriptomic analyses

Membrane spotting and analytical support were provided by the Biochips Platform (Genopole Toulouse, France). Cells were harvested from steady state continuous cultures (growth rate 0.09, 0.24, 0.35 and 0.47 h^-1^), from batch culture in exponential growth phase and after 30 min, 1.7 h and 3.5 h of BCAA starvation (isoleucine), and from batch culture in exponential growth phase and 1.6 h after norvaline addition. Cell lysis and total RNA extraction were performed as previously described [19]. RNA was quantified at 260 nm and RNA quality was controlled on electrophoresis agarose gel in denaturing conditions. Gene expression was measured using nylon arrays containing the PCR fragments (EUROGENTEC) for all genes of *L. lactis *IL1403 [16]. A constant amount of 10 μg of total RNA was used to perform the retrotranscription. Synthesis of radiolabelled cDNA, nylon arrays hybridizations and washings were performed as previously described [19]. Membranes were exposed to a phosphoimager screen for three days and scanned with a phosphofluoroimager (Storm 860, Molecular Dynamics). For each condition, three independent repetitions were performed.

### Data analyses and statistical treatment

Hybridization signals were quantified, assigned to gene names, and statistically treated with the Bioplot software (developed by S. Sokol in Plateforme Génomique, Toulouse, please see Availability & requirements for more information). Local background was removed and signals were normalized by the mean intensity of the membrane. For chemostat samples, expression ratios were calculated relative to the slowest growth rate of 0.09 h^-1^. Student tests were performed and the statistical significance of expression ratios was evaluated using Student test (p-value < 0.05) and a false discovery rate (FDR < 10%). This selection method was slightly more stringent than the one based on a FDR limit of 10%.

For isoleucine starvation and stringent response studies, expression ratios were calculated using the exponential phase as a reference. Similar statistical treatment was performed. For chemostat and isoleucine starvation data, genes whose expression is significantly modified compared to the reference in at least one of the three growth conditions have been selected in order to provide an exhaustive description of the response. Clustering analyses were performed with Genespring software (AGILENT) and R free statistical software (please see Availability & requirements for more information) was used for random forest analyses.

### Motif research

The presence of DNA pattern in un-translated region of genes has been explored using either RSAtools or MEME softwares (please see Availability & requirements for more information). In any cases these sequences were obtained from RSAtools (retrieve sequence section, default parameters).

### Data availability

Raw data of each experiment are available on GEO database (series accession number: GSE10256, GSE10254, GSE4872, GSE5761 respectively for growth rate, stringent, isoleucine starvation and glucose starvation responses).

## Results

### Growth rate response

#### Culture parameters

**Figure 1:**
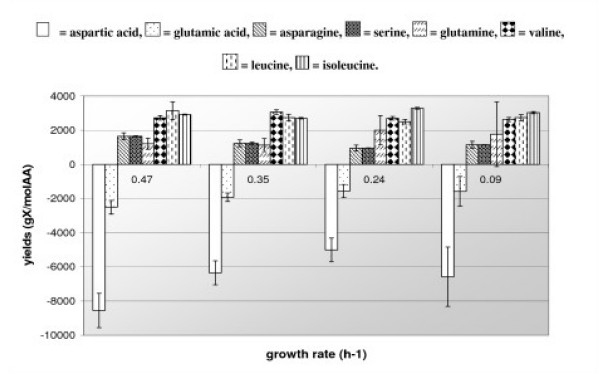
**Biomass production yields from amino acids in *L. lactis *IL1403 grown in continuous culture at different growth rates**. Amino acids with positive yield are consumed by the bacteria whereas those with negative yields are produced.

The growth rate of *Lactococcus lactis *was 5 fold increased in a chemostat culture from 0.09 to 0.47 h^-1 ^which corresponds to a decrease of the generation time from 7.7 to 1.5 h. The residual concentration of isoleucine, unlike all other amino acids, remains lower than the detection threshold (10 μM) whereas the glucose one is always high (Table 2). Biomass concentration is independent of the growth rate (Table 2) and the biomass production yield values from the different consumed amino acids are identical at the four different growth rates (Figure 1). A very weak ornithine production is observed at μ = 0.09 h^-1 ^(result not shown). The lactate residual concentration decreases when growth rate is increased (Table 2) but lactate remains the main fermentation product. The lactate production yield is indeed constant in the different growth conditions and the average value of 1.7 ± 0.1 mol of lactate per mol of glucose consumed is close to the maximum theoretical yield of 2 lactates per glucose. The production of formate, acetate and ethanol is independent of the growth rate and never exceeds 10% of this lactate production (result not shown).

**Table 2:** Macrokinetic parameters of *L. lactis *continuous cultures calculated from at least 12 different samples collected at the different growth rates.

D (h^-1^)	0.09	0.24	0.35	0.47
[X] (g/L)	0.76 +/- 0.02	0.79 +/- 0.01	0.75 +/- 0.01	0.75 +/- 0.01
[isoleucine] (μM)	<10	<10	<10	<10
[glucose] (mM)	43.23 +/- 4	70.9 +/- 2	81.05 +/- 5	84.41 +/- 2
[lactate] (mM)	116.52 +/- 6	77.57 +/- 4	57.12 +/- 6	51.85 +/- 2
Y_Gluc, Lact_ (mol Lact/mol Gluc)	1.7	1.8	1.7	1.6
qGluc (mmol/gX/h)	8.28 +/- 1	12.99 +/- 1	15.81 +/- 2	20.25 +/- 1
νLact (mmol/gX/h)	13.33 +/- 1	22.47 +/-1	25.42 +/- 3	31.54 +/-1

D = dilution rate, [ ] = residual concentration, X = biomass, Y_Gluc, Lact _= lactate yield from glucose, qGluc = specific glucose consumption rate, νLact = specific lactate production rate.

The relationship between glucose consumption rate and dilution rate is linear pointing out that the maintenance energy coefficient is constant and independent of the growth rate (5 mmol_Gluc_/gX/h).

#### Transcriptional response

As described in material and method, the slowest steady state (μ = 0.09 h^-1^) was chosen for the reference for transcriptomic analysis. Expression ratios between the various growth rates and this reference have been calculated (0,24/0,09 ; 0,35/0,09 and 0,47/0,09). Raw data are available on GEO database (GSE10256). Functional analysis was based on Bolotin *et al*. classification [16]. The over- or under-expressed genes enrichment in the various (sub) categories was determined at each steady state by Wilcoxon test on the whole category (p-value < 0.05 for significance). Main results corresponding to functional category tendencies are described in the following sections and individual genes are discussed when the statistical criteria on the gene are filled in (Student test p-value < 0.05 and FDR < 0.1).

When growth rate increases, the categories fatty acid and phospholipid metabolism (*accB, C, D, acpA, fabD, F, G1, Z1, Z2, plsX *and *yeaG*), cell division (*ezrA, ftsA, H, gid *and *rodA*) and transcription (*rnhA*, *B *(RNA degradation), *nus*B, *G, rpoA, B, C, D, E *and *smpB*) are significantly up-regulated. A wide up-regulation is also observed for the category translation (Wilcoxon p-value < 10^-6^). The expression of various genes involved in translation process is indeed increased: 6 genes encoding amino-acyl tRNA synthetase (*argS, asnS, ileS, metS, pheT *and *tyrS*), 3 genes whose products are involved in protein degradation (*pepC, T *and *yuhB*), 4 genes encoding translation factors (*infA, B, rbfA *and *tsf*) and 44 genes encoding ribosomal proteins (*gatB, prmA, rnpA, rplA, B, M, N, O, Q, S, T, U, V, X, rpmA, B, D, E, F, GB, I, J, rpsA, B, C, D, E, F, G, H, I, J, K, L, N, O, Q, R, S, T, U*).

The category energy metabolism is mostly down-regulated when the growth rate is increased according to Wilcoxon test. Nevertheless control of genes related to this category differs between the different sub categories. On one hand, genes encoding protein involved in sugar metabolism (*bglA, galM, T, gntK, Z, rbsK, scrK, uxuA, B, xylA, B, xynB *and *yidC*) are chiefly under-expressed when growth rate increases. Other genes involved in aerobic (*noxC, E, poxL, yddB, ymgK, ypgB yphA, yrjB, C, yxdE*), anaerobic (*dhaL, M, glpD*) and amino acids and amines (*arcA, B, ipd, pdc, yciA, yjiB*) metabolisms are also under-expressed. On the other hand, RNA messengers encoding proteins involved in tricarboxylic acids metabolism (*citC, D, E *and *F*) and ATP conversion (*atpB, E, F, G *and *H*) are mostly over-expressed when growth rate increases and some genes encoding glycolytic enzymes (*enoA, gapA, B, pgk, pgmB, pmg, pyk, tpiA, yjhF*) are also over-expressed.

Regarding purine and pyrimidine metabolisms, the expression of genes encoding proteins involved in interconversion of nucleotide and nucleoside (*rmlA, B, C*) globally increases under growth acceleration whereas, unexpectedly, those encoding enzymes from purine biosynthesis pathway (*purD, E,, L, M, N*) are mostly under-expressed. Genes involved in pyrimidine metabolism do not seem to be affected in a specific manner by growth rate variations (no significant Wilcoxon p-value).

The category named other is strongly down-regulated (Wilcoxon p-value < 10^-15^). Expression of phages and prophages related genes (63 *pi *and *ps*), and genes encoding transposition proteins (*tra981C, 983L, 1077B, yajE, ybdK *and *L*) are mainly under-expressed. Growth rate increase also reduces the expression of genes classified in the category transport and binding proteins and notably for the subclasses multi-drug resistance, carbohydrates, organic alcohols and acids, cations and anions.

No general tendency can be observed for the category amino acids biosynthesis: genes of histidine family (*hisC, G, Z*, and *D*) are mainly under-expressed whilst those of branched amino acids family (*ilvB, D, N, leuC *and *D*) are mostly over-expressed when growth rate is increased. More particularly some genes of the glutamate family (*argB, D, J, proC*) are under expressed. Lastly, the two categories, regulatory and unknown functions, are significantly down-regulated.

**Figure 2:**
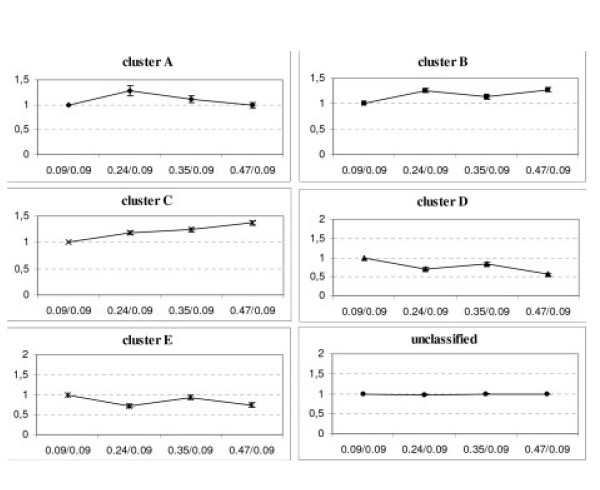
**Average expression profiles of genes classified in the different clusters during the *L. lactis *IL1403 continuous culture at different growth rate**. X axis: compared growth rate; Y axis: average expression ratios; μ = 0.09 h^-1 ^is considered as the reference. Error bars represent the 5% confidence interval.

Genes whose expression is significantly modified compared to the reference (μ = 0.09 h^-1^) in at least one of the three growth conditions have been selected with usual statistical criteria (student test p-value < 0.05 and FDR < 10%). The expression of 722 genes (377 up- and 345 down-regulations) varied significantly with the growth rate increase. These genes have been clustered with GeneSpring software in five different groups (see Figure 2 for average expression ratios). Cluster A, B and C enclose respectively 115, 149 and 169 mRNA over-expressed at every dilution rates. Genes of cluster A display a maximum over-expression at 0.24 h^-1 ^and expression ratios tend to decrease when growth rate further increases. In cluster B, over-expression increases with the growth rate though an inflexion is observed in the curve at μ = 0.35 h^-1^. Cluster C includes transcripts whose expression variations follow growth rate evolution. For genes ranked in this cluster, the biggest the difference between growth rate and the reference, the more important is the over-expression. This cluster encloses the main part of mRNA related to the translation (*argS, metS, pepC, T, pheT, prfB, rbfA, tyrS *and 35 genes encoding ribosomal proteins) and transcription (*nusG, rnhA, B, rpoA, C, E *and *smpB*) functions. Cluster D (199 genes) and E (138 genes) enclose the genes under-expressed when the growth rate increases. A stronger under-expression is observed in cluster D compared to cluster E at high growth rate. This cluster D includes transcripts belonging mainly to the category named other with more specifically 34 phages or prophages related genes (*pi *and *ps*) and 5 related to transposition (*tra981C, 983L, 1077B, ybdK, ybdL*). Moreover 31 mRNA encoding transporters and 6 transcripts belonging to the subclass sugar of the energy metabolism functional category (*bglA, galM, rbsK, scrK, xynB, yidC*) are also included in this cluster D.

#### Determinants of the growth rate response

Pattern research has been carried out in un-translated regions (upstream and downstream) of the genes belonging to the five different clusters using both RSAtools and MEME softwares. MEME identified in downstream regions a conserved pattern (5.6 × 10^-47 ^< E-value < 2.2 × 10^-5^) in each of the five groups analysed separately: gtcagtaa (or its reverse complement ttactgac). Then the frequency of the pattern has been searched in the different clusters but also in the entire genome of *L. lactis *IL1403 with RSAtools software. The average representation of the pattern in the different clusters (19.6 ± 6%) is similar to the one in the entire genome (19%) indicating that this pattern is not specifically involved in the growth rate response. One can however notice a significant under-representation of the pattern in cluster D (only 11% of genes flanked with the pattern). This result can be related to the over-representation in this cluster of genes encoding phages, prophages and transposons functions. Indeed, though the pattern is uniformly spread out in the different functional categories (21 ± 7%), a strong under-representation can be seen in the category other due to the nearly systematic lack of the pattern in the genes related to phages, prophages and transposons functions (4% of the genes). This pattern, previously identified as a highly repetitive motif [20] involved in mRNA destabilisation [19] in *L. lactis *can thus correspond to a specific feature of *L. lactis *genes; its absence could, at the opposite, be a sign of recent genetic acquisition.

**Figure 3:**
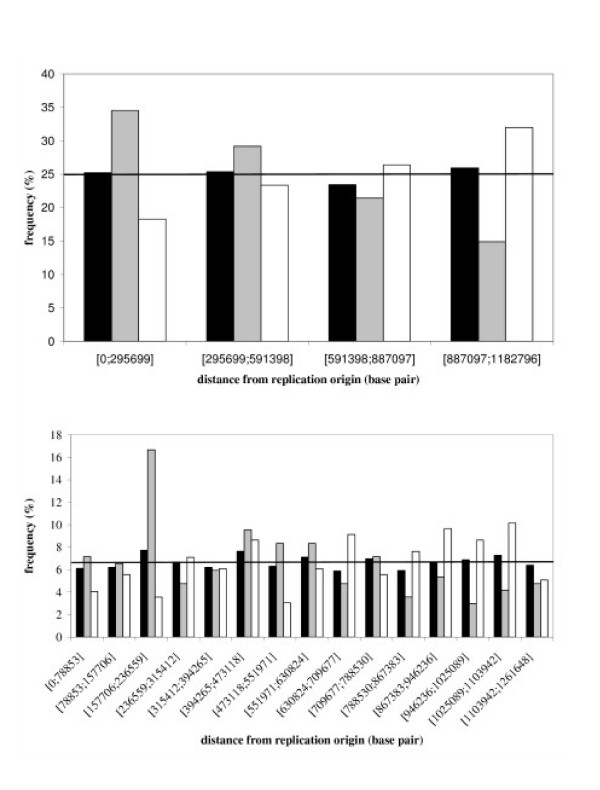
**Distribution of genes from clusters C and D along *Lactococcus lactis *IL1403 chromosome**. Dark bars indicate the global distribution of genes along the chromosome and the straight lines represent the normal one. Light grey and white bars are respectively representatives of the chromosomal localization of genes belonging to clusters C and D. The chromosome has been divided in 4 (upper graph) and 15 (lower graph) parts.

As transcriptional regulations could be influenced by chromosomal organisation, we evaluated the potential link between genes involved in the growth rate response and their distance from the replication origin. In order to determine whether the location of genes belonging to each cluster is similar or not to the global distribution of the genes along the chromosome, the test of Kolmogorov-Smirnov was used. For data independency requirements of the test, the genes belonging to the tested cluster were removed from the global distribution. The null hypothesis is rejected only for the clusters C and D (Kolmogorov p-value of 0.02 and 1.1%) indicating an atypical distribution of the genes belonging to these clusters. To complete these investigations, *L. lactis *IL1403 chromosome was divided into 8 equal parts starting from its origin (8 intervals of 295699 nt covering in average 266 genes). *L. lactis *unique chromosome being circular, these parts were then paired so as to congregate parts at the same distance from the replication origin. The distributions of the transcripts from clusters C and D as well as the global distribution in those intervals are reported in the Figure 3. One can notice that genes from cluster D are rather located far from the replication origin while genes from cluster C are mainly located close to it. A thinner division confirms this distribution profile and enables a better evaluation of gene locations (Figure 3).

### Growth rate impact in the different stress responses

#### Stringent response

As literature suggests stringent response to be a general mechanism used by cells to face growth rate reduction [12-14], stringent response and growth rate transcriptional responses have been compared. Stringent mechanism, firstly evidenced in *L. lactis *by Rallu *et al*. [21] was induced *via *norvaline addition. This leucine analogue that inhibits the amino-acylation of tRNA^ile ^and tRNA^leu ^has previously been used to induce the stringent response in *Bacillus subtilis *[15]. Norvaline was added during exponential growth of *L. lactis *in batch fermentation. Culture medium was similar to the one used for the characterization of growth rate response except that isoleucine and other branched chain amino-acid concentrations were two-fold increased in order to avoid any nutritional limitation. After norvaline addition, growth stopped for 45 min and then resumed at constant but slower rate (0.17 h^-1 ^compared to 0.78 h^-1 ^before norvaline addition). The main feature of the stringent response, consisting in the negative control of rRNA transcription [15,22], was confirmed here by the decrease of the rate of [^3^H]-uridine incorporation into RNA after norvaline addition (results not shown). Gene expression was measured in the exponential growth phase (reference sample) and 1.6 h after norvaline addition (biomass concentration of 0.82 g.l^-1^) and 461 differentially expressed genes representing the stringent response were identified. Raw data are available on Geo database (GSE10254).

**Figure 4:**
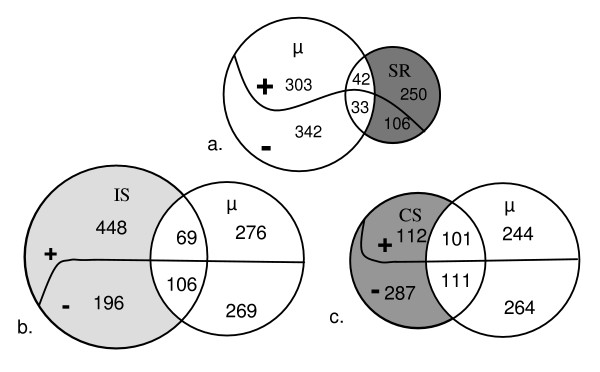
**Venn diagrams comparing the growth rate transcriptomic response with other stress responses in *L. lactis *IL1403**. Growth rate (μ) is compared with stringent response (SR) (a), isoleucine starvation (IS) (b), and carbon starvation (CS) (c). + and - respectively represents genes whose expression increases or decreases when growth rate decreases. The missing probes of the various transcriptomic datasets were removed from the diagrams.

The growth rate expression data and the stringent response have been compared with Venn diagrams. Only 75 genes were common to the stringent and growth rate responses (Figure 4a), thus revealing a weak overlap between the two responses (10% of the growth rate response). Stringent response and growth rate effect were also compared at gene functional level. The main common feature between these two responses is a generalised control of transcripts encoding translation apparatus (ribosomal proteins in essence but also transfer RNA and translation factors). However the regulation of translation machinery encoding genes seems more pronounced in continuous culture than in stringent response (57 compared to 25 genes positively regulated with growth rate) although the growth rate variations are similar in the two experiments (approximately five-fold). Similar differences are obtained with the regulation of phage related genes which is massive in continuous culture but very limited in the stringent response (63 compared to 7 genes negatively regulated with growth rate). Inversely, the stringent response pointed out a large over-expression of genes involved in stress protection but the phenomenon is more restricted in the growth rate response in continuous culture (27 and 18 genes respectively). Lastly, 88 genes are regulated in a completely opposite way in the two compared conditions.

#### Nutrient starvations

In order to evaluate growth rate impact in stress responses, the growth rate regulated genes have been compared to transcriptomic analyses obtained during glucose or isoleucine starvation. These two experiments were performed in batch cultures with the same strain, similar culture medium and environmental conditions. For isoleucine starvation experiment, growth was exponential and decelerated strongly after isoleucine was exhausted. Transcriptomic analysis was performed during the exponential growth phase and 30 min, 1.7 h and 3.5 h after isoleucine starvation. Expression ratios were calculated relative to the exponential phase. Raw data are available on GEO database (GSE4872). The carbon starvation response was previously studied in our team [23] with similar dynamic approach (GEO database, accession number : GSE5761). Transcriptomic analysis and statistical treatment were similar in these experiments compared to the chemostat study, thus allowing a rigorous comparison of the various data sets (Figure 4a and 4b). A number of 175 genes were common to isoleucine starvation and growth rate responses, which represents 24% of the whole growth rate stimulon. Similarly 212 genes accounting for 29% of the growth rate response also belong to growth and carbon starvation responses.

The global comparison between glucose, isoleucine starvations and growth rate effect shows a "core" common response including 70 genes. Among these common genes, 24 genes positively regulated by growth rate belong to the translation functional category and 3 genes negatively regulated by growth rate are phage or prophage genes. It is important to note, in this gene core, the presence of two genes encoding (putative) regulatory proteins, *llrE *and *yabB*, which are negatively regulated by growth rate.

**Figure 5:**
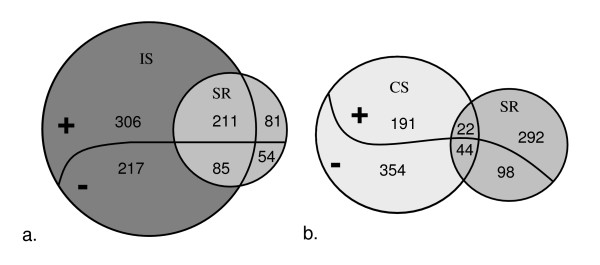
**Venn diagrams comparing the stringent transcriptomic response with other stress responses in *L. lactis *IL1403**. Stringent response (SR) is compared with isoleucine starvation (IS) response (a) and carbon starvation (CS) response (b). + and - respectively represents over- and under- expressed genes in the different responses. The missing probes of the various transcriptomic datasets were removed from the diagrams.

The two starvation conditions were also compared to the stringent response (Figure 5). The involvement of the stringent response highly differs between the two nutrient limitations though constant implication (mean 27%) has been observed above for growth rate regulation. A strong overlap between stringent response and isoleucine starvation is indeed observed (69%) while the overlap is weak with carbon starvation (14%). Furthermore 55 genes are common to the two nutrient starvations and the stringent response. However in this set of genes a strong enrichment in growth rate related genes is measured (40% of these genes belong to the growth rate response in chemostat compared to 17% obtained for the global stringent response).

#### Robustness analysis

When two or more conditions are compared, the list of (un)common genes may vary with the selection criteria initially applied on transcriptomic data (such as p-value or ratio filter), even if the same criterion is applied to all conditions. In order to test whether the overlapping percentages calculated here can depend or not on these data selection criteria, the selectivity on expression data was increased either by increasing ratio threshold or by decreasing p-value. The number of genes whose expression significantly varies in each condition decreases when the selectivity is increased, provoking a decrease in the number of transcripts in common between the different conditions (results not shown). However overlapping percentages between the responses are not strongly affected when the data selection criteria are changed (Table 3). The robustness of the comparative analysis is therefore ascertained and the conclusions drown previously can thus be reinforced.

**Table 3:** Overlapping of the different transcriptional responses with different selection criteria on datasets

**Compared conditions**	**Overlapping percentages**					
	
	**p-values < 0.05**			**ratios <1 or >1**		
		
	**ratios <1 or >1**	**ratios <0.8 or >1.2**	**ratios <0.67 or >1.5**	**p-values < 0.05**	**p-values < 0.03**	**p-values < 0.02**
μ and CS	29%	29%	31%	29%	25%	21%
μ and IS	24%	23%	20%	24%	22%	22%
μ and SR	10%	9%	5%	10%	11%	9%
SR and μ	16%	15%	8%	16%	18%	14%
SR and CS	15%	15%	17%	15%	14%	11%
SR and IS	68%	70%	72%	68%	65%	59%

Values in percentage represent the fraction of common genes compared to the whole growth rate response (for the three first lines) or the whole stringent response (for the three last lines). Abbreviations: SR = stringent response regulon, CS = carbon starvation response, IS = isoleucine starvation response, μ = growth rate response

Lastly, in order to provide a deeper analysis of the robustness of the overlapping method, other types of statistical tools (clustering or random forest analysis) have been used. The growth rate response has been discriminated into the two clusters C and D, chosen for their direct relationship with growth rate modifications, and overlaps with other responses have been estimated in each case. The overlapping of clusters C and D with carbon starvation, isoleucine starvation and stringent response are respectively 27, 31 and 15%, which confirm the previous figures. Lastly, a random forest analysis was performed on the growth rate data to select the most discriminatory genes [24], (please see Availability & requirements for more information). Unlike other statistical treatment as multiple tests, this random forest approach considers data without making any assumption on genes independency and leads to complementary results [24]. The list of discriminatory transcripts changes with every new round of random forest testing. Nevertheless, comparing the various lists with other responses gave overlapping percentages in the same order of magnitude than the previous ones.

## Discussion

### Growth rate response: metabolism

Nitrogen metabolism is crucial in lactic acid bacteria and high amino acid requirements have been described for *L. lactis *[25]. The growth of *L. lactis *IL1403 when limited by isoleucine supply in chemostat culture is characterized by homolactic metabolism. Lactate concentration remains always lower than the growth inhibiting threshold value of 150–200 mM [26,27]. This homolactic metabolism is different from the mixed metabolism observed for *L. lactis *when grown in similar conditions but with carbon limitation [26]. This result is however consistent with the work of Garrigues *et al*. [28] showing that mixed metabolism is dependant on the unbalance between catabolism and anabolism. In the chemostat limited by isoleucine supply, anabolic limitation rather than catabolic limitation is occurring. In these conditions the mixed metabolism, governed by energetic demand, is not activated due to a relative carbon flux excess. As a consequence, the metabolism remains homolactic. A deviation of the metabolism towards the mixed metabolism would have provoked a radical change in the energetic status of the cells since homolactic and mixed metabolism are not equivalent in their ATP yield. In the chemostat controlled by isoleucine supply, the energetic status of the cells is constant since maintenance and biomass yields do not vary with growth rate modifications. Isoleucine limitation and its corresponding homolactic metabolism are thus well adapted for studying the growth rate impact on cells because they avoid metabolic and energetic interferences.

### Growth rate response: transcriptomic analysis

Widespread transcriptional response is observed when growth rate is modified since more than 30% of *L. lactis *genome shows significant expression changes. Growth rate increase is accompanied by over-expression of genes involved in transcription, translation, cell division, fatty acids and phospholipids metabolism. This, and especially the massive up-regulation of 44 genes encoding ribosomal proteins, is consistent with the results observed for *S. cerevisiae *[7,9] and should be related to the acceleration of biogenesis at high growth rate. Unlike in the yeast, amino-acid and nucleotide biosynthesis pathways are not up-regulated in *L. lactis*. Such differences could be related to *L. lactis *multiple nutritional requirements and the presence of the various bases and amino-acids in the culture medium. Some genes involved in detoxification (aerobic metabolism) or in multi-drug resistance (transport) are under-expressed when the growth rate is increasing. However the expression of genes encoding important functions involved in stress protection such as chaperone, ATPase, proteases or general stress proteins, is not modified. Thus, the wide down-regulation of the genes involved in stress protection, which is observed in the yeast [7,9] when the growth rate is increased, does not occur in *L. lactis*. Finally, genes with unknown function are massively under expressed when growth rate is increased in the yeast [9] as well as in *L. lactis*. Though these genes do not necessarily share the same functions in the two micro-organisms, it can be ascertained that the cellular role of these genes is yet ignored in the two micro-organisms because they have been poorly studied due to their weak expression at high growth rates, conditions generally used in laboratories.

**Figure 6:**
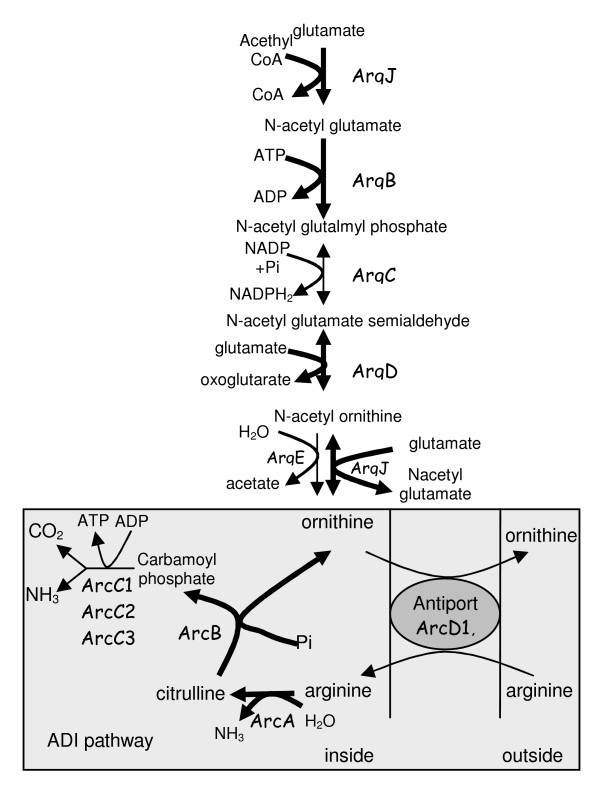
**Ornithine biosynthesis pathways in *L. lactis***. Ornithine can be synthesised from glutamate or from arginine trough the arginine deiminase pathway (ADI pathway). Thick arrows indicate up regulation of the corresponding genes upon growth rate decrease.

Genes encoding enzymes involved in ornithine biosynthesis through the arginine deiminase pathway (*arcA, arcB*) and from glutamate metabolism (*argB, argD, argJ*) are under-expressed (Figure 6) when the growth rate increases. This profile has been confirmed at macromolecular level since ornithine production, though weak, was detected at the slowest growth rate. This result raises the importance of ornithine metabolism in the growth rate adaptation of *L. lactis*. However ornithine metabolism should be disconnected from cellular energy requirements since the mixed metabolism governed by energetic demand is not activated and maintenance is also constant.

Lastly, massive under-expression of genes encoding phage and prophage related functions and transposition proteins is observed in *L. lactis *when the growth rate is increased. Phages, prophages and transposons functions can play a role in bacterial genome diversification [29]. We thus hypothesise the cells to enter, at low growth rate, a state favouring DNA rearrangements. This assumption agrees with the theory developed for *E. coli *considering that mechanisms increasing genetic adaptation through variability may provide advantages in stressful conditions [1]. The *mutX *under-expression at low growth rate could also be associated to this state favouring genetic changes since disruption of this gene homologue in *Streptococcus pneumoniae *has been proved to increase A/T to C/G transversions [30].

### Growth rate response: regulation

In order to better understand the growth rate regulations, the transcriptomic response to growth rate modifications has been compared to other transcriptomic responses through overlap calculations (Venn diagrams). The overlaps results were demonstrated to be consistent through robustness analysis since they hardly differ with the statistical data selection criteria.

Cross-comparisons of growth rate response with isoleucine and carbon starvation responses give similar overlapping percentages (mean 27%), indicating constant involvement of this mechanism in the different stress responses. The expression control of about 200 genes involved in these stress responses was thus related to growth rate modifications rather than to the stress itself. This result hence demonstrates the importance of the growth rate influence in the interpretation of all biological experiments involving growth rate decrease, *i.e*. when two environments are compared but also when two strains are analysed since mutants are most often affected in their growth capacity. Studying *E. coli *responses to nitrogen and sulphur limitations, Gyaneshwar *et al*. have found a number of genes with similar down-regulated expression profiles [31,32]. Among these common genes, some, like those encoding ribosomal proteins or those involved in fatty acids metabolism, could thus have been linked to growth rate variations. Such a comparison with the growth rate influence would have allowed a more precise characterisation of the general stress response in the yeast.

Stringent response is considered to be a general mechanism allowing cells to adjust their major physiological process to growth deceleration, independently of the growth decrease origin [12-14]. However, the comparison of growth rate and stringent responses did not reveal the deep overlap expected if these mechanisms were identical or closely related. Indeed a weak overlap (10% of the growth rate response) was obtained though the growth rate changes were similar in the two conditions. Moreover, more discrepancies (88 genes) than consistencies (75 genes) have been observed between the two responses. Functional analysis also revealed strong differences between the two responses and the under-regulation of translation function, which is considered as the main trait of the stringent response [13], is even deeper extended in the growth rate response than in the stringent response. Thus stringent and growth rate responses correspond to two distinct mechanisms and the stringent response is not the general mechanism controlling growth rate modifications in *L. lactis*. This conclusion is also supported by the findings that stringent response overlaps with carbon starvation and isoleucine starvation responses are significantly different. With a 69% overlap between stringent and isoleucine starvation responses, we can state that stringent response is included in the response to isoleucine starvation, which is not surprising since stringent response has been firstly described in *E. coli *response to amino acids starvation [22]. However, stringent response is only slightly involved in the response of *L. lactis *to glucose starvation (14% of overlapping) though this mechanism is believed to be involved in carbon starvation in *B. subtilis *[33].

Two genes encoding (putative) regulatory proteins, *llrE *and *yabB*, belong to the growth rate core. Both are poorly studied: *llrE *encodes a regulatory protein of the OmpR family found to be involved phosphate activity regulation [34] and *yabB *encodes a putative protein that could belong to the Cro/Ci regulator family. Their expression is negatively controlled by growth rate in the various conditions. Bearing in mind that *L. lactis *lacks the alternative sigma factors which ensures, in other Gram positive bacteria, extended transcriptomic responses during stress, these two genes and their regulatory function deserve to be further explored. So far, no putative regulatory motif was identified since no specific DNA pattern could have been identified for growth rate regulated genes. Gene location on the chromosome appeared to be involved in the transcription regulation of the genes belonging to the growth rate response. This is notably the case for the genes whose expression follows the growth rate evolution. The distribution on the chromosome of this genes positively regulated by growth rate (cluster C) indicates that genes whose expression increases with growth rate are mainly located close to the replication origin. The increased number of replication forks and thus the copy number of genes located in the vicinity of replication origin may contribute to this positive response. From the data obtained for *E. coli *[35], an increase of 1.5 of the replication origin copy number can be expected between 0.09 and 0.47 h^-1^, which is closely similar to the expression changes measured in the cluster C. At the opposite, genes negatively controlled by growth rate are mostly located far away from the replication origin. A different conclusion had been drawn in *S. cerevisiae *since the genes positively and negatively regulated by growth rate had been found to be located adjacent to replication origins [9]. *L. lactis *specific organisation of the growth related genes suggests a link between replication and transcription control and opens new perspectives for gene expression studies considering that gene location on the chromosome can be an actor of expression regulation though it is generally neglected.

## Conclusion

This work of integrative biology was performed at the global level using transcriptomic analysis obtained in various growth conditions. It raised the importance of growth rate regulations and provided, for the first time in a bacterium, the full description of the growth rate response. This work also participated to the elucidation of the mechanism involved in growth rate control. Though the mechanism is not yet fully understood in *L. lactis*, one expected regulatory mechanism (the stringent response) has been ruled out, two potential regulators (*llrE *and *yabB*) have been identified. Finally the involvement of gene location on the chromosome has also been found to be involved in the expression regulation of these growth related genes, opening new perspectives for gene expression studies.

## Availability & requirements

Bioplot software: 

R free statistical software: 

RSAtools: 

MEME: 

The Comprehensive R Archive Network: 

## Authors' contributions

CD, ER, PL and MC–B: conception, data acquisition, analysis of the data, drafting of the manuscript; CD, MC–B, HM and PB: statistical treatment of the data; all the authors read and approved the final manuscript.
